# Supplementary material for: Ellagic Acid Affects Metabolic and Transcriptomic Profiles and Attenuates Features of Metabolic Syndrome in Adult Male Rats
Source: Nutrients. 2021 Mar 1;13(3):804. doi: 10.3390/nu13030804 (PMC8001306; doi:10.3390/nu13030804)
Supplement: Supplementary file 1 [file nutrients-13-00804-s001.zip › 1109637-suppl/SupplTabS1S2.pdf]

SUPPLEMENTARY MATERIAL

Ellagic acid affects metabolic and transcriptomic profiles and attenuates features of metabolic syndrome in adult male rats

Adéla Kábelová (1), Hana Malínská (2), Irena Marková (2), Olena Oliyarnyk (2), Blanka Chylíková (1), Ondřej Šeda (1)

[1] Institute of Biology and Medical Genetics, the First Faculty of Medicine, Charles University and the General University Hospital, Prague, Czech Republic

[2] Institute for Clinical and Experimental Medicine, Prague, Czech Republic

Corresponding author: Ondřej Šeda

Institute of Biology and Medical Genetics

First Faculty of Medicine, Charles University,

Albertov 4, 12800 Prague 2, Czech Republic

Tel: +420 224 968 180; email: oseda@lf1.cuni.cz

Supplementary tables

Supplementary table S1. Effect of ellagic acid supplementation on oxidative stress markers in liver, kidneys and heart of HFD and HFD-EA rats. Variables are mean ± SEM, n = 6-8 for each group. GSH:GSSG; reduced:oxidized glutathione ratio, TBARS; thiobarbituric acid reactive substances, HFD; high-fat diet-fed rats, HFD-EA; high-fat diet-fed rats supplemented with ellagic acid

Oxidative stress markers	**HFD**	**HFD-EA**	**HFD x HFD-EA**
**Liver**			
Reduced glutathione (µmol/mg protein)	38.1±2	44.2±3	0.15
Oxidized glutathione (µmol/mg protein)	2.47±0.2	1.54±0.1	0.006
GSH:GSSG	16.3±1	29.4±2	0.0003
Glutathione reductase (nmol NADPH/min/mg protein)	105±5	147±17	0.037
Glutathione peroxidase (µmol NADPH/min/mg protein)	209±22	272±16	0.05
Glutathione S-transferase (nmol CDNB/min/mg)	143±11,9	156±14.19	0.54
Catalase, µmol H_2O_2 (NADPH/min/mg protein)	1636±141	2089±93	0.031
Superoxide dismutase (U/mg protein)	0.08±0.006	0.1±0.003	0.047
TBARS (nmol/mg protein)	1.88±0.2	1.33±0.1	0.028
**Kidneys**			
Reduced glutathione (µmol/mg protein)	36.2±2	44.6±4	0.08
Oxidized glutathione (µmol/mg protein)	2.98±0.3	1.78±0.1	0.002
GSH:GSSG	12.6±0.6	25.4±2	0.0002
Glutathione reductase (nmol NADPH/min/mg protein)	113±7	119±7	0.56
Glutathione peroxidase (µmol NADPH/min/mg protein)	214±10	276±21	0.025
Glutathione S-transferase (nmol CDNB/min/mg)	26.4±2	29±2	0.37

Catalase, µmol H_2O_2 (NADPH/min/mg protein)	575±27	730±32	0.004
Superoxide dismutase (U/mg protein)	0.03±0.002	0.05±0.003	0.0006
TBARS (nmol/mg protein)	0.87±0.06	0.83±0.07	0.6612
**Heart**			
Reduced glutathione (µmol/mg protein)	45.2±3	44.7±3	0.88
Oxidized glutathione (µmol/mg protein)	3.3±0.1	3.55±0.3	0.40
GSH:GSSG	13.9±0.9	12.8±0.7	0.43
Glutathione reductase (nmol NADPH/min/mg protein)	64.6±6	80.1±5	0.11
Glutathione peroxidase (µmol NADPH/min/mg protein)	108±5	99.9±5	0.32
Glutathione S-transferase (nmol CDNB/min/mg)	38.8±3	42±5	0.56
Catalase, µmol H_2O_2 (NADPH/min/mg protein)	386±22	446±28	0.13
Superoxide dismutase (U/mg protein)	0.05±0.003	0.05±0.003	0.32
TBARS (nmol/mg protein)	0.81±0.5	0.54±0.02	0.0003

Supplementary table S4. Transcripts validated by qPCR and their expression changes in brown adipose tissue in response to ellagic acid administration to SHR-*Zbtb16*$^{Lx/k.o.}$ rat males

Gene symbol	Gene name	P (HFD vs HFD-EA)	Fold change (microarray)	Fold change (qPCR)
Dio2	Iodothyronine deiodinase 2	3.04E-10	-24.35	-13.38
Gck	Glucokinase	n.s.	n.s.	n.s.
Pla2g2a	phospholipase A2, group IIA (platelets, synovial fluid)	9.43E-06	4.55	8.47
Tnxb	tenascin XB	4.68E-06	2.95	3.11
Nr4a1	nuclear receptor subfamily 4, group A, member 1	2.48E-09	-5.75	-4.65
